# Supplementary material for: Canine-Origin Platelet-Rich Fibrin as an Effective Biomaterial for Wound Healing in Domestic Cats: A Preliminary Study
Source: Vet Sci. 2021 Sep 30;8(10):213. doi: 10.3390/vetsci8100213 (PMC8539014; doi:10.3390/vetsci8100213)
Supplement: Supplementary file 1 [file vetsci-08-00213-s001.zip › vetsci-1304087-supplementary.pdf]

veterinary sciences

MDPI

Supplementary Materials

Table S1. Therapeutic schedule instituted for each cat during the PRFs' treatment period

Case	Pharmacological Treatment Instituted
#1	Meloxicam 0.1 mg/kg once a day, orally, during 5 consecutive days;<br>Amoxicillin-clavulanic acid 20 mg/kg twice a day, orally, for 8 days;<br>Methadone 0.3 mg/ kg twice a day, subcutaneously, in the 24 hours after the orthopaedic surgery;
#2	Meloxicam 0.1 mg/kg once a day, orally, during 5 consecutive days;<br>Amoxicillin-clavulanic acid 20 mg twice a day, orally, for 8 days;<br>Methadone 0.3 mg/ kg q twice a day, subcutaneously, in the 48 hours after the orthopaedic surgery;<br>Prolivet (Bioiberica) 100 mg once a day,, during 30 days;
#3	Meloxicam 0.1 mg/kg once a day, orally, during 5 consecutive days;<br>Clindamycin 8mg/kg twice a day, orally, for 10 days;
#4	Meloxicam 0.1 mg/kg once a day, orally, during 5 consecutive days;<br>Initial therapy with amoxicillin-clavulanic acid 20 mg/kg twice a day, orally, being substituted by doxycycline 10 mg/kg once a day, orally, for 28 consecutive days after *Bartonella* sp. diagnose;<br>Buprenorphine 0.02mg/kg twice a day, subcutaneously, during the initial 3 days.
